# Efficacy of Xuebijing Combined with Ulinastatin in the Treatment of Traumatic Sepsis and Effects on Inflammatory Factors and Immune Function in Patients

**DOI:** 10.3389/fsurg.2022.899753

**Published:** 2022-05-03

**Authors:** Yuanchao Su, Yunliang Zhang, Hongsheng Yuan, Chuan Shen

**Affiliations:** Department of Emergency Medicine, Chongqing Qijiang District People’s Hospital, Chongqing, China

**Keywords:** trauma, sepsis, Xuebijing, ulinastatin, inflammatory factors, immune function, liver function

## Abstract

**Objective:**

To investigate the efficacy of xuebijing combined with ulinastatin in the treatment of traumatic sepsis and analyze the effects on inflammatory factors and immune function of patients.

**Methods:**

182 patients with traumatic sepsis were selected from June 2017 to September 2021 in our hospital. The patients were divided into the control group and the observation group. Patients in both groups were given routine treatments such as initial resuscitation, blood transfusion, monitoring of lactic acid to guide fluid replacement, early control of infection source, selection of appropriate antibiotics, correction of acidosis, treatment of primary disease, prevention of hypothermia and stress ulcer, application of vasoactive drugs, application of glucocorticoid and nutritional support. The control group was treated with Xuebijing injection on the basis of routine treatment, and the observation group was given Xuebijing injection combined with ulinastatin treatment on the basis of routine treatment. The APACHE II score was applied to evaluate the patients before and after treatment, and the routine blood indicators, inflammatory factor indicators, immune function indicators and liver function indicators were tested.

**Results:**

After the treatment, the APACHE II score of the observation group was (10.35 ± 3.04) lower than that of the control group (15.93 ± 4.52) (*P* < 0.05). After treatment, the WBC and neutrophils in the observation group (15.19 ± 2.91) and (0.65 ± 0.04) were lower than those in the control group (16.42 ± 3.44) and (0.79 ± 0.05), and the PLT(162.85 ± 43.92) was higher than that in the control group (122.68 ± 36.89) (*P* < 0.05). After treatment, the levels of serum PCT, IL-6, TNF-α in the observation group were (11.38 ± 3.05), (10.74 ± 3.82) and (9.82 ± 2.35) lower than those in the control groups (17.34 ± 3.29), (15.28 ± 4.05) and (13.24 ± 3.06) (*P* < 0.05). After treatment, the levels of CD3+, CD4+, CD8+, CD4+/CD8+ in the observation group were (50.64 ± 4.98), (40.56 ± 4.82), (27.22 ± 3.29), (1.49 ± 0.24) higher than those in the control groups (46.08 ± 4.75), (34.69 ± 4.08), (25.14 ± 3.18), (1.38 ± 0.19) (*P* < 0.05). After treatment, the levels of TBIL and AST in the observation group were (12.35 ± 3.82), (25.66 ± 4.49) lower than those in the control group (18.43 ± 4.06), (34.58 ± 5.06) (*P* < 0.05).

**Conclusion:**

Xubijing combined with ulinastatin has a good effect in the treatment of patients with traumatic sepsis, which can effectively improve the condition, reduce the body’s inflammatory response, and promote the recovery of patients’ immune function and liver function.

## Introduction

With the development of economy and modern transportation, the incidence of trauma increases year by year, and the injury condition is increasingly complex and serious. Sepsis is a syndrome caused by the body’s malfunctioning response to an infection. The infection range can affect the whole body, and it is often the common complication after severe trauma, critical illness, infection and major surgery. It usually causes functional damage to important tissues and organs (liver, kidney, lung), and further deterioration will lead to tissue hypoperfusion and continuous hypotension, which in turn develops into severe sepsis and septic shock (1–4). Sepsis has a very important connection with trauma. Sepsis is one of the most serious complications in trauma patients, which easily leads to multiple organ dysfunction syndrome. Sepsis is not only an excessive inflammatory response, but also the result of immune dysfunction that interacts with the inflammatory and anti-inflammatory processes (5, 6). Studies have shown that the onset of traumatic sepsis is likely to lead to systemic organ function damage, and the liver is the most vulnerable organ to inflammatory injury. 0.6%∼50.0% of patients with traumatic sepsis will suffer from sepsis-related liver injury (7, 8). Therefore, effective prevention and treatment of liver function impairment plays an important role in the prognosis of patients with traumatic sepsis. At present, there is no very effective method for the treatment of sepsis, which is mainly based on the pathogenesis of sepsis, and comprehensive support treatments for its pathogenesis are mainly given, such as early fluid resuscitation, control of infection, mechanical ventilation, maintaining the stability of organ function, hormone therapy, but its mortality rate has not improved (9, 10). Xuebijing Injection has the effects of activating blood, resolving stasis, clearing heat, detoxicating, and strengthening the body resistance. It is currently commonly used for the treatment of patients with sepsis (11, 12). Ulinastatin is effective for alleviating inflammation and regulating immune function, and its role in the clinical treatment of sepsis has become increasingly prominent (13, 14). The purpose of this study was to investigate the efficacy of Xuebijing combined with ulinastatin in the treatment of traumatic sepsis and analyze its effects on the inflammatory factors and immune function of patients.

## Materials and Methods

### Patients

Total of 182 patients with traumatic sepsis were selected from June 2017 to September 2021 in our hospital. Inclusion criteria: All the patients met the relevant diagnostic criteria for traumatic sepsis in the 2012 International Guidelines for the Diagnosis and Treatment of Severe Sepsis and Septic Shock (15); They were all the first confirmed cases of traumatic sepsis; There was no previous history of Xuebijing or ulinastatin treatment. Exclusion criteria: Patients with active bleeding and unable to stop bleeding or other serious diseases that cannot be controlled; Patients with diabetes, tumor and other basic diseases; Patients with contraindications to drugs in this study; Recent history of glucocorticoid treatment; People whose conditions can’t be controlled. The patients were divided into the control group and the observation group, 91 cases in each group. There was no significant difference in general data between the two groups (*P* < 0.05). As shown in **Table 1**.

**Table 1:** Comparison of general data between the two groups.

Groups	Gender		Age(years)	APACHE II score(points)	SOFA score(points)
	Male	Female			
Control group (*n* = 91)	53	38	53.08 ± 7.16	22.08 ± 5.92	12.49 ± 2.85
Observation group (*n* = 91)	50	41	52.76 ± 7.32	22.24 ± 5.86	12.68 ± 2.93
t/χ^2^	0.201		0.298	0.183	0.443
*P*	0.654		0.766	0.855	0.658

### Treatment Methods

Patients in both groups were given routine treatments such as initial resuscitation, blood transfusion, monitoring of lactic acid to guide fluid replacement, early control of infection source, selection of appropriate antibiotics, correction of acidosis, treatment of primary disease, prevention of hypothermia and stress ulcer, application of vasoactive drugs, application of glucocorticoid and nutritional support. The control group was treated with Xuebijing injection (Produced by Tianjin Hongri Pharmaceutical Co., Ltd., Batch number: 20180317) on the basis of routine treatment. 50 mL of Xuebijing injection was added into 100 mL of sodium chloride injection, and intravenous infusion was completed, twice a day. For patients with severe disease, the treatment was given three times a day. On the basis of routine treatment, the observation group was treated with Xuebijing injection combined with ulinastatin (Produced by Guangdong Tianpu Biochemical Medicine Co., LTD., Batch Number: 20171019). The medication method of Xuebijing injection was the same as that of the control group. The ulinastatin for injection was added into 100 mL of sodium chloride injection at 300,000 units each time and given by intravenous infusion twice a day. The treatment lasted for seven days in both groups.

### Observation indicators

#### Acute Physiological and Chronic Health Status Scoring System II(APACHE II) Score Detection

The APACHE II score was applied to evaluate patients before and after treatment, respectively (16). The scale totally included acute physiological score, age score and chronic health score. The higher the score was, the worse the prognosis was.

#### Detection of Blood Routine Indexes

5 mL of venous blood was collected from patients, and blood leukocytes (WBC), platelets (PLT) and neutrophils were detected using XFA automatic blood cell analyzer (Produced by Beijing Jiapukang Biotechnology Co., LTD.).

#### Detection of Inflammatory Factors

ELISA was used to detect the levels of procalcitonin (PCT), interleukin-6(IL-6) and tumor necrosis factor-α (TNF-α). The kit was purchased from Adlitteram Diagnostic Laboratories, and the microplate reader was Anthos 2010.

#### Detection of Immune Function Indicators

T-lymphocyte subsets (CD3+, CD4+, CD8+, CD4+/CD8+) were measured using FACSCount flow cytometer produced by BD Company in the United States and supporting reagents.

#### Detection of Liver Function Indicators

Total bilirubin (TBIL) was detected by thrombin method before and after treatment, and aspartate aminotransferase (AST) was detected by oxaloacetate dehydrogenase method.

#### Adverse Reactions

The incidence of adverse reactions during treatment was recorded.

### Statistical Methods

The results of this experiment were statistically analyzed by SPSS 20.0 (SPSS Co., Ltd., Chicago, USA). Measurement data were expressed by (mean ± standard deviation), and t test was used for their comparison between groups. *P* < 0.05 indicates that the difference is statistically significant.

## Results

### Comparison of APACHE II Scores

**Figure 1:**
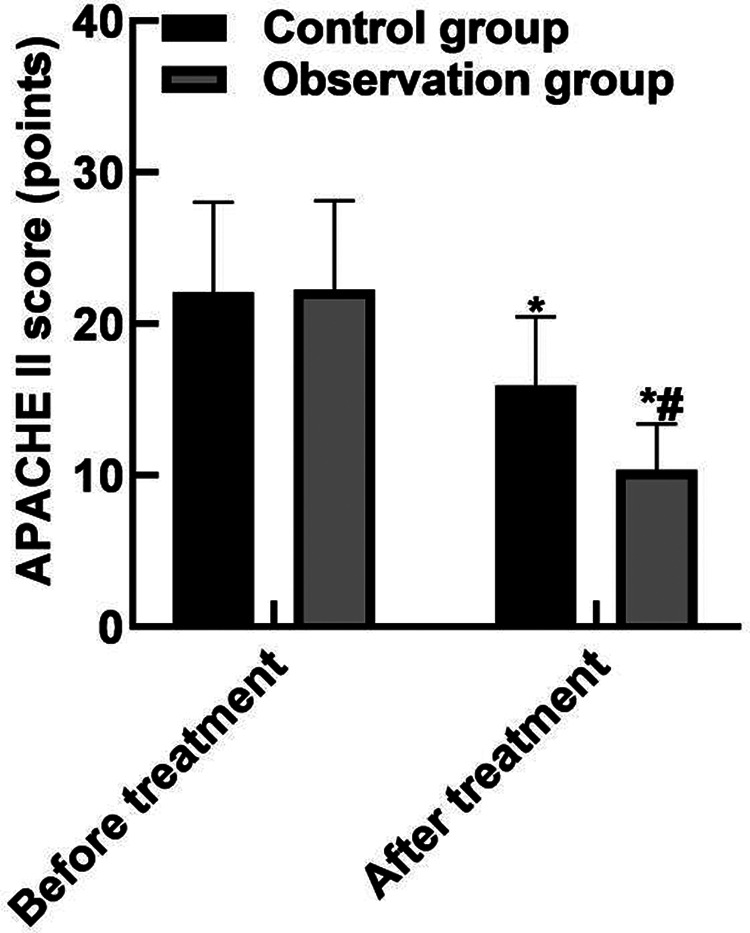
Comparison of APACHE II scores. Note: Compared with before treatment, *P < 0.05; Compared with the control group, ^#^*P* < 0.05.

After the treatment, the APACHE II score in the observation group was (10.35 ± 3.04) lower than that in the control group (15.93 ± 4.52) (*P* < 0.05). As shown in **Figure 1**.

### Comparison of Blood Routine Indexes

**Figure 2:**
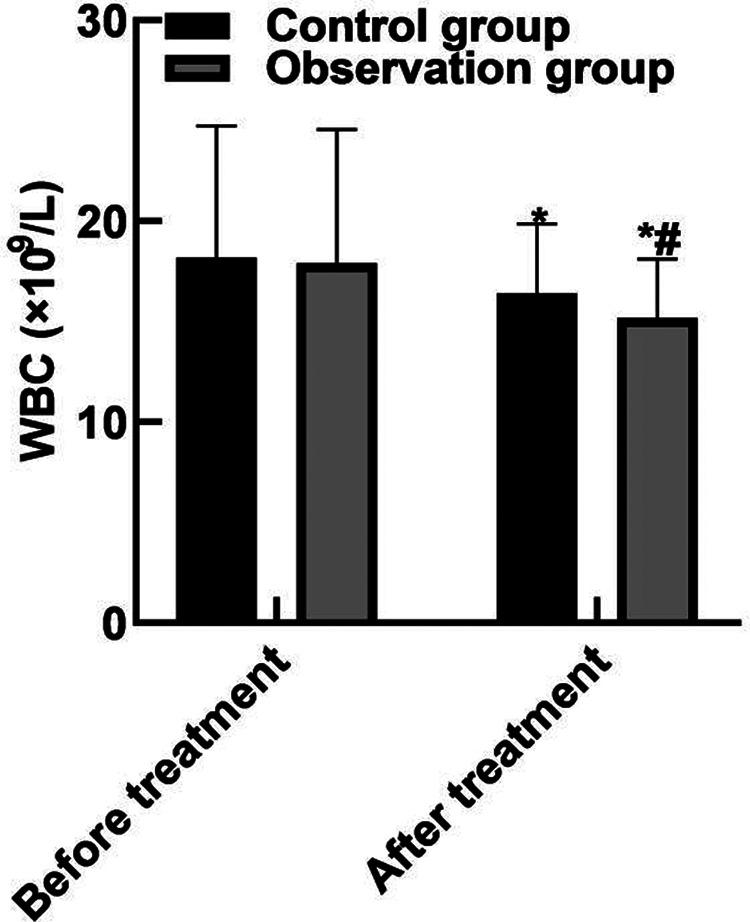
Comparison of blood routine indexes. Note: Compared with before treatment, *P < 0.05; Compared with the control group, ^#^*P* < 0.05.

After treatment, the WBC and neutrophils in the observation group were (15.19 ± 2.91) and (0.65 ± 0.04) lower than those in the control group (16.42 ± 3.44) and (0.79 ± 0.05), and the PLT(162.85 ± 43.92) was higher than that in the control group (122.68 ± 36.89) (*P* < 0.05). As shown in **Figure 2**.

### Comparison of Inflammatory Factors

**Figure 3:**
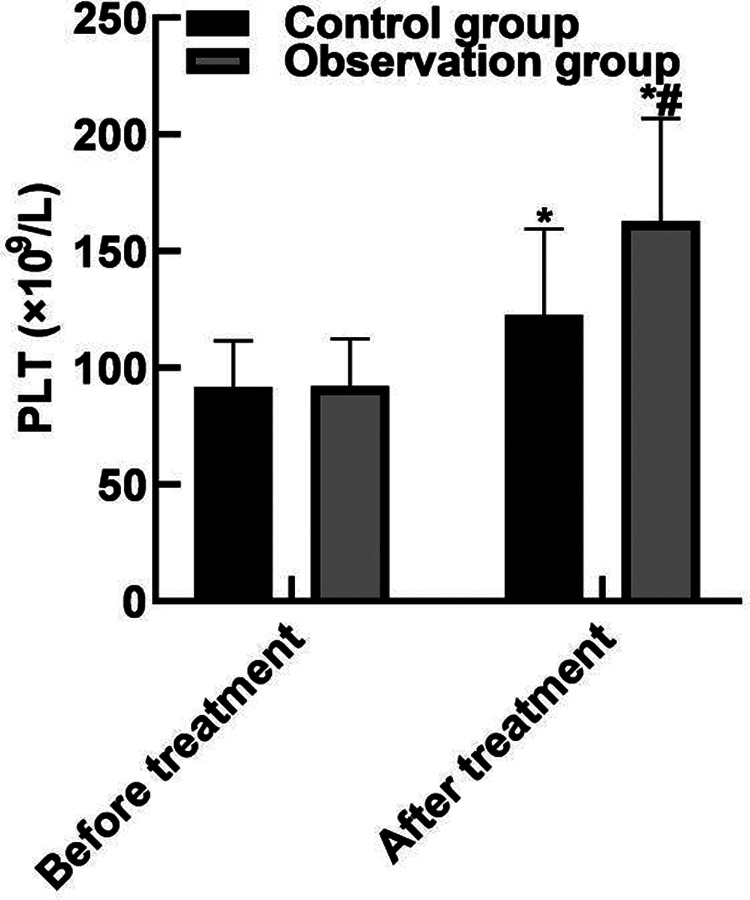
Comparison of inflammatory factors. Note: Compared with before treatment, *P < 0.05; Compared with the control group, ^#^*P* < 0.05.

After treatment, the levels of serum PCT, IL-6, TNF-α in the observation group were (11.38 ± 3.05), (10.74 ± 3.82) and (9.82 ± 2.35) lower than those in the control groups (17.34 ± 3.29), (15.28 ± 4.05) and (13.24 ± 3.06) (*P* < 0.05). As shown in **Figure 3**.

### Comparison of Immune Function Indicators

**Figure 4:**
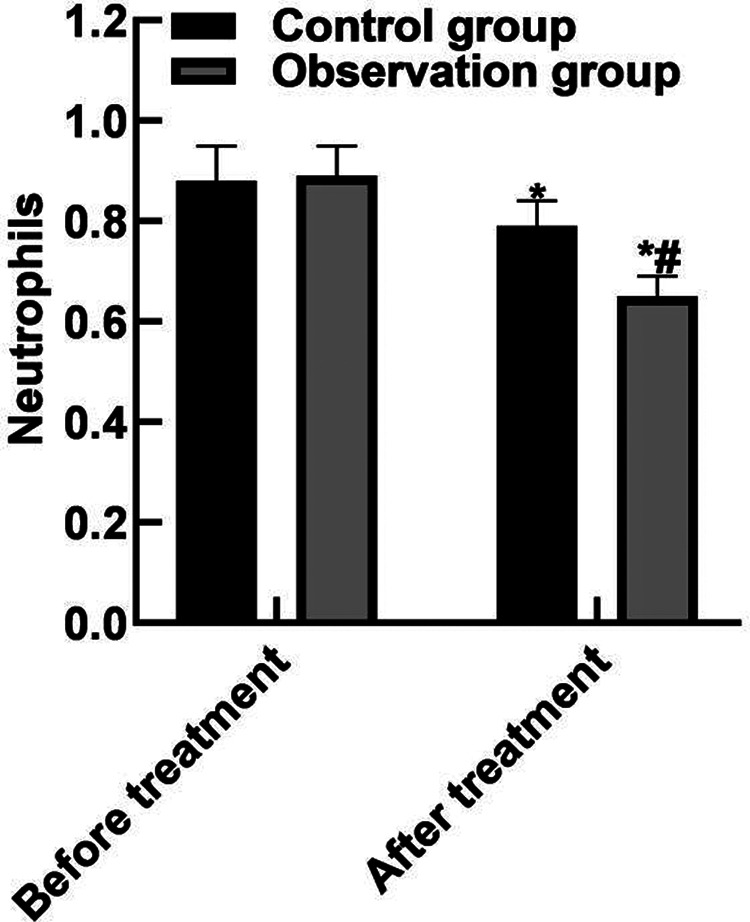
Comparison of immune function indicators. Note: Compared with before treatment, *P < 0.05; Compared with the control group, ^#^*P* < 0.05.

After treatment, the levels of serum CD3+, CD4+, CD8+, CD4+/CD8+ in the observation group were (50.64 ± 4.98), (40.56 ± 4.82), (27.22 ± 3.29), (1.49 ± 0.24) higher than those in the control groups (46.08 ± 4.75), (34.69 ± 4.08), (25.14 ± 3.18), (1.38 ± 0.19) (*P* < 0.05). As shown in **Figure 4**.

### Comparison of Liver Function Indicators

**Figure 5:**
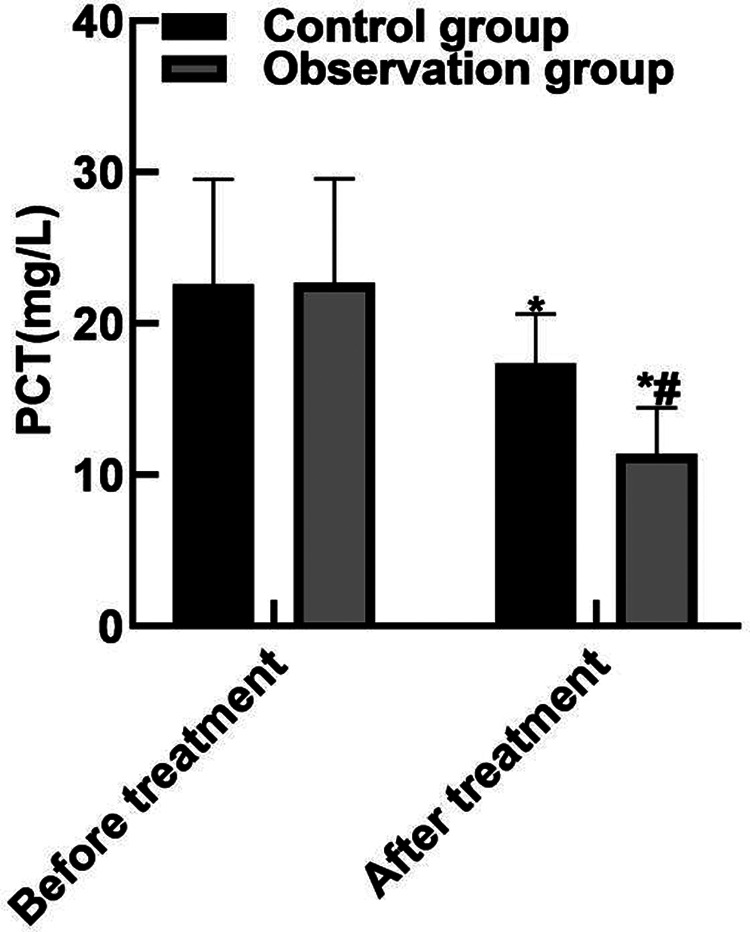
Comparison of liver function indicators. Note: Compared with before treatment, *P < 0.05; Compared with the control group, ^#^*P* < 0.05.

After treatment, the levels of TBIL and AST in the observation group were (12.35 ± 3.82), (25.66 ± 4.49) lower than those in the control group (18.43 ± 4.06), (34.58 ± 5.06) (*P* < 0.05). As shown in **Figure 5**.

### Comparison of Adverse Reactions

During the treatment, paroxysmal atrial fibrillation occurred in 1 case, gastrointestinal reaction in 2 cases and rash in 3 cases in the control group, and the incidence of adverse reactions was 5.49% (5/91). In the observation group, paroxysmal atrial fibrillation occurred in 2 cases, gastrointestinal reaction in 2 cases, rash in 4 cases, and the incidence of adverse reactions was 8.79% (8/91). There was no significant difference in the incidence of adverse reactions between the two groups (*P* > 0.05).

## Discussion

The infection of patients with traumatic sepsis can come from wounds, surgical sites, lungs, blood, urinary tract and other areas. Any open wound can easily become the source of bacterial colonization and infection, and the surgical incision and surgical site are also prone to occur, especially when there is contamination surgery such as internal fixation implantation or intestinal cavity opening (17, 18). Patients with severe trauma often have respiratory impairment, especially in patients with severe craniocerebral injury, chest trauma that cannot rule out airway secretions and the use of mechanical ventilation. Pulmonary infection has become the main source of traumatic sepsis (19, 20). Decreased immunity in trauma patients is an intrinsic cause of susceptibility to infection, including local and systemic defense deficits. Among them, much attention has been paid to the functional decline of neutrophils, mononuclear macrophages, lymphocytes, and dendritic cells, which are considered to be the factors occupying an important position in the pathogenesis of sepsis, manifested as decreased serum opsonin, decreased granulocyte generation, inhibition of neutrophil chemical tropism and bactericidal ability, and defects of specific immune responses, such as reduced lymphocyte formation (21, 22). Trauma induces inflammatory response and also produces a series of anti-inflammatory factors, which inhibit the binding of inflammatory factors to receptors on the cell membrane and down-regulate the immune response. Anti-inflammation and pro-inflammation interact, leading to immune regulation disorders or even low immunity (23, 24). Therefore, effective reduction of inflammatory response and recovery of immune function are the important basis for the treatment of sepsis. The occurrence of sepsis is directly related to the severity of trauma. Patients with severe trauma should be sent to medical institutions with corresponding treatment capacity as soon as possible and receive definitive treatment from professionals as soon as possible. This can reduce the occurrence of sepsis and organ dysfunction and reduce the mortality rate (25, 26). Xuebijing injection has the effects of promoting blood circulation, removing blood stasis, clearing away heat and detoxification and strengthening the root. Many pharmacological studies have shown that Xuebijing Injection has the effects of improving coagulation function, microcirculation and protecting vascular endothelial cells. At the same time, it can prevent secondary injury of liver and other organs by inhibiting the release of inflammatory factors and blocking the cascade waterfall reaction of inflammatory factors (27, 28). The defense system of the human body is mainly completed by nerve regulation and hormone. When the body is hit hard, it can stimulate the stress response and promote the release of inflammatory cell mediators, thus damaging tissue cells. Ulinastatin has the effects of stabilizing lysosomal enzymes and inhibiting the release of inflammatory cells, which can reduce inflammation, protect internal organs, fight infection and remove oxygen free radicals (29, 30).

The results of this study showed that after the treatment, the APACHE II score of the observation group was (10.35 ± 3.04) lower than that of the control group (15.93 ± 4.52), the WBC and neutrophils of the observation group were (15.19 ± 2.91) and (0.65 ± 0.04) lower than those of the control group (16.42 ± 3.44) and (0.79 ± 0.05), and the PLT was (162.85 ± 43.92) higher than that of the control group (122.68 ± 36.89). These results indicated that Xuebijing combined with ulinastatin could improve the condition and prognosis of patients with traumatic sepsis. Analysis of the reasons are as follows: Xubijing can effectively antagonize endotoxin and inflammatory mediators, protect vascular endothelial cells, improve coagulation function, dilate blood vessels, improve microcirculation and tissue perfusion, reduce capillary permeability, promote fiber tissue reabsorption and repair of tissue lesions, regulate immunity, and protect tissues and organs. And ulinastatin is a broad spectrum of protease inhibitors, can stabilize lysosomal membrane and membrane, restrain various proteolytic enzyme activity, inhibiting inflammatory factor, the production of oxygen free radical and release, also can protect the vascular endothelial cells, adjust the blood coagulation and immune function, improve microcirculation and tissue perfusion, thus reduce tissue damage. Chen (31) believed that TNF-α is the initiator of inflammatory response and the key mediator of the damaging effect of endotoxin. TNF-α induces the production of inflammatory cytokines such as IL-6, activates lymphocytes and a variety of inflammatory transduction pathways, participates in the immune response, and finally affects organ functions. Il-6 co-promotes T lymphocyte proliferation with TNF-α. Besides, the results of this study showed that the levels of serum PCT, IL-6, and TNF-α in the observation group after treatment were (11.38 ± 3.05), (10.74 ± 3.82), (9.82 ± 2.35) lower than those in the control groups (17.34 ± 3.29), (15.28 ± 4.05), (13.24 ± 3.06). These results indicated that Xuebijing combined with ulinastatin could reduce the inflammatory response in patients with traumatic sepsis. The reason was analyzed that the main components of Xuebijing Injection could improve microcirculation and increase blood flow. Reduce that inflammatory reaction and the permeability of capillaries, reduce inflammatory exudation, promoting the absorption of inflammation, and inhibiting the formation of inflammatory granulomas, thereby sufficiently reduce the inflammatory reaction. Ulinastatin can inhibit trypsin, kallikrein and neutrophil elastase, and has a significant blocking effect on the activation of inflammatory cells and cascade cascade reaction between inflammatory factors. In addition, ulinastatin can improve microcirculation and tissue perfusion, thereby further improving the inflammatory response of patients.

The results of this study showed that after treatment, the serum levels of CD3+, CD4+, CD8+, and CD4+/CD8+ in the observation group were (50.64 ± 4.98), (40.56 ± 4.82), (27.22 ± 3.29), (1.49 ± 0.24) higher than those in the control group (46.08 ± 4.75), (34.69 ± 4.08), (25.14 ± 3.18), (1.38 ± 0.19). These results indicated that Xuebijing combined with ulinastatin could restore the immune function of patients with traumatic sepsis. The reason was analyzed that Xuebijing Injection combined with ulinastatin could improve the immune function decline, protein metabolism abnormalities and renal function decline induced by infection stimulation, prevent the organ and cell damage induced by endotoxin stimulation, and improve the microcirculation of patients during shock. The liver is the most vulnerable organ to inflammatory injury. The production of excessive free radicals and the ischemia and hypoxia of the body can lead to damaged membrane protein function and the destruction of mitochondrial membrane and liver membrane, thus causing damage to the secretion, uptake and transport of bilirubin by hepatocytes. Acute liver injury may be caused by sepsis at any stage, mainly manifested as elevated transaminases and TBIL and coagulation disorders. The results of this study also showed that after treatment, the levels of TBIL and AST in the observation group were (12.35 ± 3.82), (25.66 ± 4.49) lower than those in the control groups (18.43 ± 4.06), (34.58 ± 5.06). The reason was analyzed as follows: Xuebijing injection can inhibit the release of inflammatory factors and improve the synthesis ability of liver protein, thus reducing liver injury; Moreover, it has protective effect on liver, which may be related to regulating p-STAT3 signaling pathway and inhibiting the overexpression of pro-inflammatory factors. Ulinastatin can improve microcirculation and remove oxygen free radicals, fundamentally control cell damage and protect the liver function of patients.

## Conclusion

Xubijing combined with ulinastatin has a good effect in the treatment of patients with traumatic sepsis, which can effectively improve the condition, reduce the body’s inflammatory response, and promote the recovery of patients’ immune function and liver function.

## Data Availability

The original contributions presented in the study are included in the article/supplementary material, further inquiries can be directed to the corresponding author/s.
